# Comparative Analysis of Silicone Mouth Swabs with Varying Hardness Levels for Optimal Plaque Removal in Elderly Oral Care

**DOI:** 10.3290/j.ohpd.b5866440

**Published:** 2024-12-02

**Authors:** Nutthawadee Engsomboon, Bhornsawan Thanathornwong, Siriwan Suebnukarn

**Affiliations:** a Nutthawadee Engsomboon Lecturer, Faculty of Dentistry, Srinakharinwirot University, Bangkok, Thailand. Data curation, formal analysis, investigation, methodology, project administration.; b Bhornsawan Thanathornwong Associate Professor, Faculty of Dentistry, Srinakharinwirot University, Bangkok, Thailand. Conceptu-alisation, data curation, formal analysis, methodology, project administration, writing (original draft), review and editing.; c Siriwan Suebnukarn Professor, Faculty of Dentistry, Thammasat University, Pathum Thani, Thailand. Conceptualisation, data curation, formal analysis, investigation, methodology, supervision, writing (original draft), review and editing.

**Keywords:** dentistry, dental hygiene, oral health, silicone

## Abstract

**Purpose:**

Silicone mouth swabs have emerged as a promising alternative to gauze, sponge brushes, and soft-bristled toothbrushes, offering a balance between gentle cleaning and effectiveness. The flexibility and softness of silicone make it a suitable material for safely cleaning the sensitive oral tissues of elderly patients. This study aims to determine the optimal hardness level of silicone that maximises cleaning effectiveness while minimising the risk of trauma to oral tissues.

**Materials and Methods:**

A pseudo-plaque was created by mixing 6.0 g of Thicken Up Clear food additive with 12.0 ml of water and food colouring, which was then spread onto a NISSIN dentoform silicone rubber sheet (simulated soft tissue) with a thickness of 2.0 mm. Silicone heads with different hardness levels – 20, 30, 40, 50, and 60 Shore A – were attached to a V.P.2000 tooth brushing machine, operating at 75 rounds per minute with a force of 1.96 N. Each swab was used to brush the surface 25 times (n = 16 for each group).

**Results**: A one-way analysis of variance (ANOVA) revealed a statistically significant difference in pseudo-plaque removal among the five hardness levels, with an F-value of 106.161 (degrees of freedom = 4, 75, p < 0.001). The Games-Howell pairwise comparison test showed that all five silicone hardness levels differed significantly from each other in their effectiveness in removing pseudo-plaque (p < 0.05). No visible simulated soft tissue damage was observed before and after brushing, as inspected with a stereomicroscope in all experiments.

**Conclusion:**

The silicone oral swab with a hardness level of 60 Shore A was found to maximise pseudo-plaque removal *in vitro*. This finding is crucial for the development of specialised oral hygiene tools tailored to the needs of the elderly population, thereby enhancing oral health and overall well-being.

As the global population ages, the demand for effective elderly care continues to rise, bringing significant challenges to healthcare systems worldwide. According to the World Health Organization (WHO), the proportion of people aged 60 and older is expected to nearly double from 12% to 22% between 2015 and 2050.^9^ This demographic shift underscores the urgent need for comprehensive healthcare solutions tailored to the unique needs of the elderly. Among the myriad health concerns faced by this population, maintaining proper oral hygiene stands out as a critical aspect that directly impacts overall health and quality of life.^5^

Oral hygiene in elderly care is particularly challenging due to the prevalence of conditions such as reduced manual dexterity, cognitive impairments, and an increased risk of oral diseases like periodontal disease and dental caries.^1^ These challenges necessitate the development of specialised tools and techniques to ensure effective oral care, which is vital not only for preventing oral infections but also for avoiding systemic health issues such as cardiovascular diseases and aspiration pneumonia.^7^ Traditional oral hygiene tools, such as gauze, sponge brushes, and soft-bristled toothbrushes, have been widely used in this population. However, these tools often present challenges, such as inadequate cleaning efficiency or the potential to cause trauma to delicate oral tissues.^11^

Silicone mouth swabs have emerged as a promising alternative, offering a balance between gentle cleaning and effectiveness.^4^ The flexibility and softness of silicone make it a suitable material for safely cleaning the sensitive oral tissues of elderly patients. However, the hardness of silicone, typically measured by a durometer, can significantly influence the swab’s cleaning efficiency and its ability to remove dental plaque.^3^ Harder silicone may provide more effective plaque removal, while softer silicone might be better suited for individuals with sensitive oral tissues. Despite the critical role of these tools, there has been limited research into the impact of silicone hardness on the efficacy of plaque removal. This gap in research is particularly relevant in elderly oral care, where the balance between gentleness and effective plaque removal is critical.

This study aims to address this gap by conducting a comparative analysis of silicone mouth swabs with different hardness levels. By evaluating the plaque removal efficiency of swabs with varying durometer readings, this research aims to determine the optimal hardness level that maximises cleaning effectiveness while minimising the risk of trauma to oral tissues. The hypothesis posited that the silicone oral swab with a hardness level of 60 Shore A would maximise pseudo-plaque removal *in vitro*. This information is vital for the development of specialised oral hygiene tools that can better serve the needs of the elderly population, ultimately contributing to improved oral health and overall well-being.

## MATERIALS AND METHODS

This experimental study aimed to compare the effectiveness of silicone mouth swab heads with varying hardness levels – 20, 30, 40, 50, and 60 Shore A – in removing dental plaque. A silicone mouth swab head, designed by our research group, features both straight and threaded brushing bristles to enhance access to different areas of the mouth, including challenging spots like the back of the oral vestibule and underneath the tongue. Sample size determination was carried out using G*Power version 3.1. Based on an effect size of f = 0.4, a power of 0.8, and an alpha level of 0.05, the required sample size was calculated to be 80, divided into 5 groups of 16 samples each. The effectiveness of each silicone mouth swab was evaluated by analysing and calculating the area of pseudo-plaque removed after brushing.

### Silicone Mouth Swab

**Fig 1:**
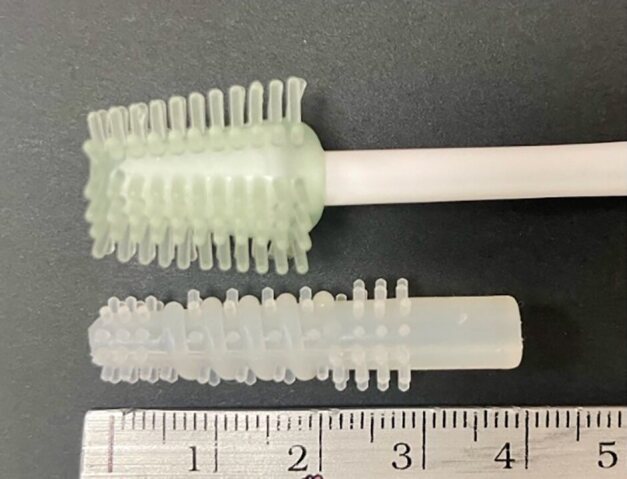
The MouthEze (MC3, Oral Care Innovations, United Kingdom) (top) and a newly designed silicone mouth swab (bottom).

Our team developed a newly designed silicone mouth swab as a modification of the MouthEze (MC3, Oral Care Innovations, United Kingdom). The MouthEze (MC3, Oral Care Innovations, UK) is a specialised oral hygiene tool designed for gentle and effective cleaning of the mouth, particularly for individuals who face challenges using traditional toothbrushes. It is widely used in elderly care, hospitals, and by caregivers for patients with limited dexterity, cognitive impairments, or other conditions that make routine oral hygiene difficult. The MC3 features a silicone head, specifically engineered to be soft and gentle on the delicate tissues of the mouth. The silicone material is less abrasive than traditional bristles, thereby reducing the risk of trauma to sensitive areas such as the gums, tongue, and oral mucosa. The MC3’s straight bristles are designed to clean surfaces effectively without causing irritation (Fig 1, left).

The new design was tailored with straight and threaded brushing bristles to better access different areas of the mouth, including hard-to-reach spots like the back of the oral vestibule and underneath the tongue (Fig 1, right). The length of the swab head was increased to ensure it could reach all necessary areas while maintaining control and precision. The silicone used in the new swab is medical grade, chosen for its comfort and flexibility.

### Brushing Simulation

**Fig 2:**
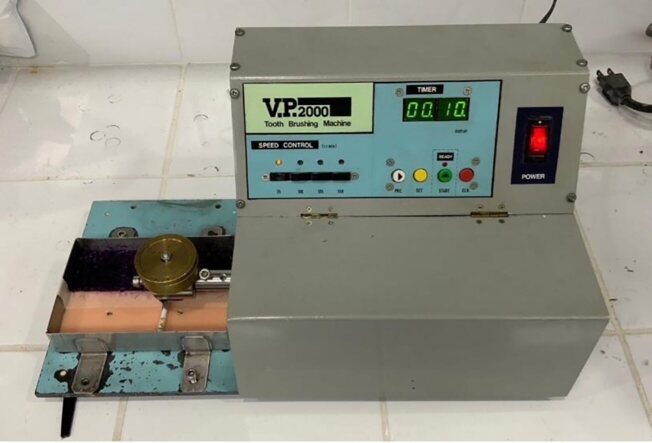
The V.P.2000 toothbrushing machine.

The V.P.2000 toothbrushing machine was used in this study (Fig 2). The V.P.2000 is a specialised laboratory device used to simulate the mechanical action of brushing in controlled experimental settings. It is designed to provide consistent and repeatable brushing conditions for testing the abrasion resistance, cleaning efficiency, and durability of dental materials and oral hygiene tools. The machine can operate at various speeds, typically ranging from 75 to 150 rotations per minute (rpm). The V.P.2000 is equipped with a system to apply specific weights, usually in the range of 50–500 g, to mimic the pressure exerted during brushing. The V.P.2000 typically simulates a back-and-forth brushing motion, which is common in manual toothbrushing. The machine is commonly used to test the wear resistance of dental materials, such as sealants, fillings, and crowns, by simulating prolonged exposure to brushing. Researchers use the V.P.2000 to assess the effectiveness of different toothbrushes, swabs, and other oral hygiene tools in removing plaque or simulated biofilm from dental surfaces. The machine provides a standardised environment for testing, ensuring that results are not influenced by variations in brushing technique or force, which can occur with manual testing.

**Fig 3:**
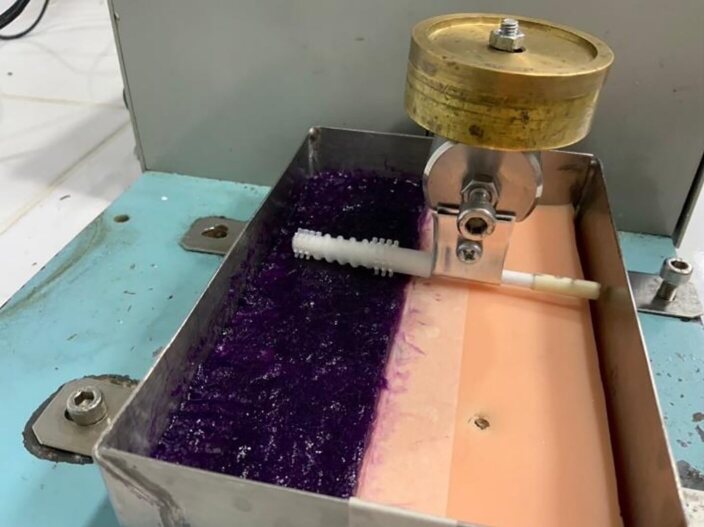
The silicone head was attached to a V.P.2000 toothbrushing machine.

A pseudo-plaque, created by mixing 6.0 g of Thicken Up Clear food additive with 12.0 ml of water and food colouring, was spread onto a NISSIN dentoform silicone rubber sheet (simulated soft tissue) with a thickness of 2.0 mm. The silicone head with different hardness levels (20, 30, 40, 50, and 60 Shore A) was attached to a V.P.2000 toothbrushing machine, operating at 75 rounds per minute with a force of 1.96 N, and each was used to brush the surface 25 times (Fig 3).

### Outcome Measures and Statistical Analysis

**Fig 4:**
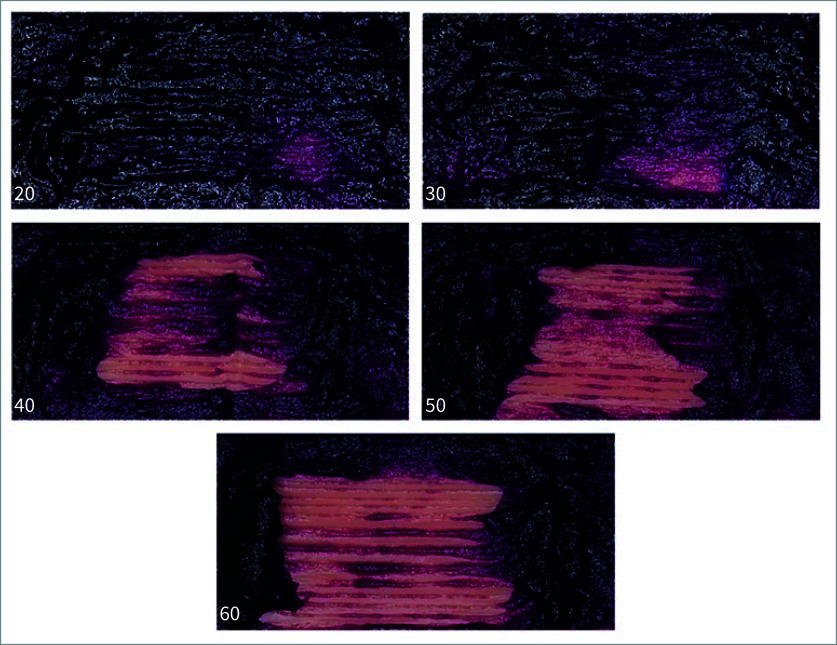
The pseudo-plaque removal images for the silicone mouth swab varying hardness levels – 20, 30, 40, 50, and 60 Shore A – after brushing.

The ImageJ programme was used to analyse and quantify the area where the pseudo-plaque was removed after brushing. ImageJ is an open-source image processing programme widely used in scientific research for analysing and measuring various types of images. Originally developed at the National Institutes of Health (NIH), ImageJ allows researchers to perform complex image analysis with high precision. The programme is especially popular in fields like biology, medicine, and material science for tasks such as measuring areas, counting objects, and analysing spatial distribution. In this study, ImageJ was used to analyse and measure the area where pseudo-plaque was removed after brushing under both wet and dry conditions. After brushing, the simulated soft tissue was photographed using an Olympus digital camera PEN Lite E-PL5 (F3.5, 1/60 shutter speed, ISO 320) (Fig 4). The process of image analysis using the ImageJ programme included setting the scale of the image for accurate analysis, enhancing the visibility of the pseudo-plaque removal area, converting the image to 8-bit for processing, setting the threshold, converting the image to binary, creating a mask, and eroding the area for calculation. The final result represents the quantified pseudo-plaque removal area. Determination of simulated soft tissue damage after brushing was performed using a stereomicroscope to capture detailed images of the silicone soft tissues before and after brushing.

The outcome measures in this study include the area where pseudo-plaque was removed after brushing. A one-way analysis of variance (ANOVA) was conducted to assess differences in pseudo-plaque removal. Further pairwise comparisons using the Games-Howell test were conducted to assess the difference in each pair of silicone hardness levels. Statistical analysis was performed by IBM SPSS version 22 (SPSS, Chicago, IL, USA, 2022). Values of p < 0.05 were considered statistically significant.

## RESULTS

The pseudo-plaque removal images for the silicone mouth swab varying hardness levels (20, 30, 40, 50, and 60 Shore A) after brushing are shown in Figure 4. There were no visual signs of wear or damage, such as surface abrasions, indentations, or thinning of the simulated soft tissues inspected with the stereomicroscope in all experiments. Table 1 presents the area of pseudo-plaque removal after brushing. A one-way ANOVA was conducted to compare the effectiveness of five different levels of silicone hardness (20, 30, 40, 50, and 60) in the removal of pseudo-plaque. The analysis revealed a statistically significant difference among the five hardness levels, with an F-value of 106.161, degrees of freedom (df) = 4, 75, and a p-value of 0.000. Further pairwise comparisons using the Games-Howell test revealed that all five silicone hardness levels (20, 30, 40, 50, and 60) differ significantly from each other in terms of their effectiveness in removing pseudo-plaque, with a significance level of 0.05 (Table 2).

**Table 1:** The area of pseudo-plaque removal after brushing with the silicone mouth swab varying hardness levels –20, 30, 40, 50, and 60 Shore A

Silicone mouth swab hardness levels (Shore A)	n	Mean area of pseudo-plaque removal after brushing (mm^2^)	Standard deviation	Standard error	95% Confidence interval
Upper bound	Lower bound
20	16	62,188.94	839.67	209.91	57,478.61	66,899.27
30	16	85,669.94	291.97	247.99	80,878.45	90,461.42
40	16	152,460.75	335.38	258.84	134,857.43	170,064.07
50	16	252,090.81	1,481.45	1,270.36	224,658.28	279,523.34
60	16	452,684.81	2,900.9	2,725.22	387,195.55	518,174.08


**Table 2:** Pairwise comparisons using the Games-Howell test to assess the difference in each pair of silicone hardness levels

Silicone mouth swab hardness levels (Shore A)	Silicone mouth swab hardness levels (Shore A)	Mean difference	Standard error	95% Confidence interval
Upper bound	Lower bound
20	30	–23,481.000*	3,152.336	–32,624.86	–14,337.14
	40	–90,271.813*	8,549.404	–116,260.11	–64,283.51
	50	–189,901.875*	13,058.714	–229,944.12	–149,859.63
	60	–390,495.875*	30,804.597	–485,495.22	–295,496.5
30	20	23,481.000*	3,152.336	14,337.14	32,624.86
	40	–66,790.813*	8,559.325	–92,797.05	–40,784.58
	50	–166,420.875*	13,065.211	–206,473.86	–126,367.89
	60	–367,014.875*	30,807.352	–462,018.50	–272,011.25
40	20	90,271.813*	8,549.404	64,283.51	116,260.11
	30	66,790.813*	8,559.325	40,784.58	92,797.05
	50	–99,630.063*	15,292.313	–144,467.92	–54,792.21
	60	–300,224.063*	31,815.845	–396,925.27	–203,522.86
50	20	189,901.875*	13,058.714	149,859.63	229,944.12
	30	166,420.875*	13,065.211	126,367.89	206,473.86
	40	99,630.063*	15,292.313	54,792.21	144,467.92
	60	–200,594.000*	33,311.946	–300,226.16	–100,961.84
60	20	390,495.875*	30,804.597	295,496.53	485,495.22
	30	367,014.875*	30,807.352	272,011.25	462,018.50
	40	300,224.063*	31,815.845	203,522.86	396,925.27
	50	200,594.000*	33,311.946	100,961.84	300,226.16
* The mean difference was significant at p < 0.001.

## DISCUSSION

Silicone brushes have been developed^2^ as alternatives to tools currently used to clean oral soft tissues in older adults, including gauze, sponge brushes, soft-bristled brushes, and damp cleaning cloths.^4^ Among these, sponge brushes are the most popular, but they have notable drawbacks, including lower effectiveness in cleaning the mouth and reducing microbial biofilm. Additionally, there is a risk of the sponge brush heads detaching and causing choking.^6^ Soft-bristled brushes, when used improperly with excessive force, can cause undesirable effects such as excessive abrasion of the oral mucosa. To address these issues, in healthcare, particularly in oral hygiene care, the selection of silicone material is critical due to its direct interaction with delicate oral tissues. Medical-grade silicone is commonly used because it is biocompatible, hypoallergenic, and free from toxic chemicals, ensuring it does not cause irritation or adverse reactions when used in the mouth. The hardness of silicone, measured by the Shore A durometer scale, plays a significant role in determining its functionality in oral care tools. The Shore A scale is typically used for softer, flexible materials like those found in oral hygiene products. A balance must be struck where the silicone is firm enough to effectively remove plaque without causing trauma to sensitive oral tissues. This study utilised silicone with varying hardness levels, ranging from 20 to 60 Shore A, to identify the optimal hardness that maximises cleaning efficiency while ensuring safety for elderly patients. The findings underscore the importance of precise material selection in the design of oral hygiene tools that cater to the unique needs of this population.

Despite the widespread use of products like MouthEze (MC3) silicone brush, there is a notable gap in existing research concerning the optimisation of oral hygiene tools specifically designed for cleaning soft tissues. While MC3 and similar tools are commonly used, evidence of their efficacy is limited, highlighting the need for innovations in this area. Our study addressed this gap by introducing a new silicone mouth swab featuring both straight and threaded brushing bristles, along with a longer head length of 24.0 mm, compared to the MC3 swab, which has only straight bristles and an 18.0 mm head. By evaluating the plaque removal efficiency of swabs with varying hardness levels, this research sought to determine the optimal hardness level that maximises cleaning effectiveness while minimising the risk of trauma to oral tissues. The results supported our hypothesis that the silicone oral swab with a hardness level of 60 Shore A would maximise pseudo-plaque removal *in vitro*. No visible simulated soft tissue damage was observed before and after brushing, as inspected with a stereomicroscope in all experiments.

The cleaning equipment used in this study was the V.P.2000 toothbrushing machine, which operates at a constant speed. This machine can be set to various speeds – 75, 100, 125, and 150 rotations per minute (rpm) – and is equipped with weights of 50, 100, and 150 g. According to the research conducted by Sangpanya and colleagues, the V.P.2000 toothbrushing machine was utilised to test the abrasion resistance of dental sealants. In their study, the machine simulated back-and-forth brushing with a 300-g weight and performed 48,000 brushing strokes.^8^ In addition, research by Wiegand and colleagues compared the force exerted during manual brushing to that of electric brushing. They found that manual brushing typically exerts an average force of 1.6 ± 0.3 Newtons.^10^ Based on these findings, our study used a weight of 200 g on the V.P.2000 toothbrushing machine to simulate realistic brushing conditions.

It is important to acknowledge the limitations of our study. As an* in-vitro* study using the V.P.2000 toothbrushing machine, our simulation was limited to a single plane of brushing action. In real-world scenarios, brushing involves multiple directions and planes, which our study did not replicate. To further validate the effectiveness of our silicone mouth swab, future research should include studies with phantom heads and human participants. These studies should involve both self-cleaning and caregiver-assisted cleaning to better reflect real-life conditions and to ensure the broad applicability of our findings.

## CONCLUSION

The silicone oral swab with a hardness level of 60 Shore A was found to maximise pseudo-plaque removal *in vitro*. This finding is crucial for the development of specialised oral hygiene tools tailored to the needs of the elderly population, thereby enhancing oral health and overall well-being.
